# Clinical Characteristics, Risk Factors, and Predictors of Fatal Outcomes and Prolonged Hospitalization of Crimean–Congo Hemorrhagic Fever Cases in Basrah, Iraq

**DOI:** 10.3390/idr18030049

**Published:** 2026-05-19

**Authors:** Mohammed H. Al-Maliki, Celine Tabche, Alaa K. Mousa, Ali R. Hashim, Zeenah Atwan, Hassan A. Farid, Maitham G. Yousif, David Rawaf, Nazik Haikaz Hasrat, Murtadha Almusafer, Anees K. Nile, Riyadh Al-Hilfi, Azeem Majeed, Alessandra Scagliarini, Salman Rawaf, Roaa Khafaji, Juan Carlos de la Torre, Haydar Witwit

**Affiliations:** 1 Basrah Teaching Hospital, Basrah Health Directorate, Basrah 61004, Iraq; 2 WHO Collaborating Centre for Public Health Education and Training, School of Public Health, Department of Primary Care and Public Health, Imperial College London, London W12 0BZ, UK; z.atwan@imperial.ac.uk (Z.A.);; 3 Department of Internal Medicine, College of Medicine and Basrah Teaching Hospital, University of Basrah, Basrah 61004, Iraq; 4 Central Laboratory, Microbiology Department, College of Medicine, University of Basrah, Basrah 61004, Iraq; 5 Manchester Centre for Clinical Neurosciences, Salford Royal Hospital, Salford, Greater Manchester M6 8HD, UK; 6 College of Science, Al-Qadisiyah University, Al Diwaniyah 58001, Iraq; 7 Training and Human Development Centre, Basrah Health Directorate, Basrah 61001, Iraq; 8 Department of Surgery, College of Medicine, University of Basrah, Basrah 61004, Iraq; 9 College of Medicine, Al-Nahrain University, Alkadhimiya, P.O. Box 70076, Baghdad 10072, Iraq; 10 Directorate of Public Health, Ministry of Health, Baghdad 10001, Iraq; 11 Department of Medical and Surgical Sciences, University of Bologna, 40126 Bologna, Italy; 12 Independent Researcher, Chula Vista, CA 91910, USA; dr.r.khafaji@gmail.com; 13 Department of Immunology and Microbiology, The Scripps Research Institute, La Jolla, CA 92037, USA

**Keywords:** Crimean–Congo hemorrhagic fever (CCHF), disease outbreak, Iraq, clinical presentation, infectious diseases

## Abstract

Background: The impact of climate change on birds’ migration and ticks’ reservoir habits is contributing to the spread of Crimean–Congo hemorrhagic fever (CCHF), caused by CCHF virus (CCHFV), to new continents and countries. CCHF is endemic to the Eastern Mediterranean Region, including Iraq, and is witnessing a substantial surge in confirmed cases with considerable disparity and gaps in managing CCHF cases. The increasing CCHF spread across Asia, Africa, and Europe, including Spain and Turkey, highlights the danger of its expansion. Developing high-confidence diagnostic criteria, identifying risk factors, and accurate predictors of CCHF outcomes are critical to managing suspected and confirmed cases of CCHF and to reducing the current case fatality rate of CCHF, which is the goal of this study. Methods: We completed a retrospective evaluation of 61 confirmed cases of CCHF in Basrah (Iraq). The cases were screened according to the clinical presentation, and CCHF cases were identified by ELISA and validated by PCR. Data was analyzed using SPSS version 22. T-tests, chi-square/Fisher exact tests, and Pearson’s correlation were used, with significance set at *p* < 0.05 and high significance at *p* < 0.01. Results: We found that repeated exposure to animals during animal slaughtering was a significant risk factor. In addition, 5% of the patients with confirmed CCHF, mainly from rural areas, reported exposure to rats. Clinical presentations included fever, headache, gastrointestinal problems, eye and orbital symptoms, and hemorrhagic complications. Predictors of death included advanced age, decreased platelet counts, and neuropsychiatric symptoms such as delusions and confusion. Conclusions: Our findings identify clinical and laboratory features of CCHF cases in Iraq, which will help to implement the most effective interventions to manage CCHF cases and protect the public in all Iraqi governorates. In summary, this study highlights a recent and significant rise in CCHF cases in Basrah Governorate, Iraq. Notably, 5% of confirmed cases reported contact with rats. The paper also proposes diagnostic criteria and identifies key predictors of mortality to support improved clinical management of CCHF. These findings underscore the urgent need for strengthened public health interventions, including enhanced infection prevention and control measures, increased awareness, and improved surveillance systems. The findings have important implications for improving control procedures, guiding therapeutic development, informing vaccine strategies, and supporting evidence-based policy alongside future research efforts.

## 1. Introduction

Crimean–Congo hemorrhagic fever (CCHF) is one of the most common vector-borne diseases spreading through Asia, Africa, and Europe. CCHF virus (CCHFV), the causative agent of CCHF, was discovered separately in the Crimea Peninsula in the 1940s and the Republic of Democratic Congo in the 1960s [1,2,3,4]. CCHFV (*Orthonairovirus* genus, Nairoviridae family, Bunyavirales order) is an enveloped arbovirus with a tri-segmented negative-sense RNA genome [5].

CCHFV has been spreading widely with reports of cases in Turkey, Iran, India, Greece, the Republic of Georgia, some Balkan states, and more in Spain [6]. CCHF incidence has surged in the Eastern Mediterranean Region (EMR), but no accurate data exists about the magnitude and implications of this surge [7].

CCHFV reservoirs and vectors are tick species, mainly *Hyalomma marginatum*. The virus is transmitted through a tick bite or contact with infected animals or people. CCHFV is maintained in a silent enzootic tick-vertebrate-tick cycle. Vertebrates contribute to CCHFV epidemiology in three ways: (1) providing blood meals that allow immature ticks to develop, (2) transporting ticks across geographic borders through animal movement or migration, and (3) serving as a source of viremia that transmits CCHFV to feeding ticks (transstadial transmission) and directly to humans [1,8]. Various domestic and peri-domestic animals can be asymptomatically infected and contribute to human transmission through direct contact with infected blood or tissues (including undercooked meat), posing a significant risk to individuals and workers in close contact with livestock, such as butchers, farmers, and veterinarians, but also to healthcare professionals when proper protective disposal and procedure are not employed during delivering healthcare to CCHF patients [9,10]. The epidemiological importance of birds is still debated, but avian species frequently host *Hyalomma* spp. and can facilitate the spread and the expansion of tick habitat, raising concern about the consequent possible CCHFV spreading [11]. Accordingly, CCHFV has been documented in ticks carried by migratory birds [12,13,14]; however, their potential role as reservoirs has not been investigated extensively [15].

Two main mechanisms explain the bleeding symptoms in CCHF: (1) direct viral effects on endothelial cells and thrombocytes, and (2) cellular damage caused by the host’s inflammatory response [16]. Activated endothelial cells produce proinflammatory cytokines, especially Interleukin 6 IL-6 and IL-8, both of which promote permeability of vessels and vasodilatation, hypotension, multiple organ failure, shock, and probably death; on the other hand, CCHFV can also impair innate and adaptive immune responses [11,17].

The CCHF incubation period depends on the transmission route and viral load, and it usually takes a few days to one week. The shorter incubation period is associated with tick bites, whereas exposure to viremic livestock and nosocomial infections is associated with longer incubation periods [18]. The outcome of CCHFV infection ranges from asymptomatic to mild or severe clinical manifestations leading to death. About 88% of infections are subclinical; tracking them is critical for assessing the protective role of herd immunity [19] and to minimize the risk of spread in places where the zoonotic lifecycle is maintained. Early symptoms are similar in fatal and non-fatal cases, but cases with fatal outcomes develop later common clinical features, including ecchymosis, hematemesis, melena, somnolence, and gingival bleeding. In addition, laboratory parameters including lower platelet count, prolonged prothrombin time (PT) and higher alanine aminotransferase (ALT), aspartate aminotransferase (AST), lactate dehydrogenase (LDH), and creatine phosphokinase (CPK) levels, are associated with high fatality risk. Thrombocytopenia, prolonged activated partial thromboplastin time (aPTT), and melena and somnolence are strong predictors of mortality [20].

In Iraq, CCHF was first reported in 1979 [21,22]. Two further outbreaks occurred in 1992 and 1996, followed by a steady increase from 1998 to 2009. In 2022, there were 219 PCR-confirmed cases and 518 in 2023 [23,24,25]. The reasons for the rise in CCHF cases in 2022 and 2023 are not well understood. Alterations in agricultural practices and increased human–animal interactions may have heightened exposure to vectors of CCHFV. However, the disruption of pest control measures during the COVID-19 pandemic might have been a contributing factor [23]. Likewise, pandemic-related restrictions and resource limitations constrained veterinary services, disrupting routine acaricidal spraying and ectoparasite control programs, which likely contributed to the proliferation of the tick population [26]. Basrah governorate is in the far south of Iraq. It is a large city with an urban center and rural areas at the periphery of the center. The region has a hot desert climate with temperatures exceeding those recorded in other areas of Iraq. The highest recorded average temperature was 47.47 °C in August 2023, whereas in 2022 it was 43.74 °C in June [27]. Despite these elevated temperatures, the types of cultivated crops and farm animals are comparable to those found in other parts of southern Iraq. The increased number of confirmed CCHF cases in Iraq prompted this study to establish high-confidence diagnostic criteria based on clinical features and defined laboratory tests, identify predictors of fatal outcomes, and develop recommendations for public health intervention. The conclusions when disseminated could result in a unified and improved management of CCHF cases.

## 2. Materials and Methods

### 2.1. Type of Study and Location

A retrospective observational cohort study was conducted at the infectious diseases unit of Basrah Teaching Hospital in Basrah City, Southern Iraq.

### 2.2. Data

The study included de-identified records of all patients with laboratory-confirmed CCHFV infection treated at Basrah Teaching Hospital between 1 May 2022 and 1 August 2023. Confirmed cases were defined by detection of CCHFV RNA by PCR. Serologic testing by ELISA (Catalog No. abx157316, IgG, Abbexa, Cambridge, UK) was used as complementary laboratory evidence when available, but acute infection was established by PCR positivity. PCR was performed using the RealStar^®^ CCHFV RT-PCR Kit 1.0 (Order No. 181013, altona DIAGNOSTICS, Hamburg, Germany) according to the manufacturer’s instructions, and amplification signals were detected using the CFX96 Real-Time System (Bio-Rad, Hercules, CA, USA). Blood samples were collected in heparinized, sodium citrate gel tubes for complete blood count, coagulation and bleeding profile, renal and liver function tests, and inflammatory markers using Roche Cobas Integra 400 plus and Roche Cobas C311 clinical chemistry analyzers (Roche, Indianapolis, IN, USA). The samples were processed in a biosafety level 2 cabinet. Comprehensive contact histories with animals and infected individuals were obtained, and probable transmission routes were assessed. All the clinical data were recorded, including shared and non-shared symptoms between fatal and non-fatal cases. Complete blood counts, bleeding profiles, and biochemical tests were assessed at admission and discharge.

### 2.3. Population

A total of 61 patients were identified with CCHF disease.

### 2.4. Data Processing and Analysis

Records were de-identified, coded, and analyzed using SPSS version 22 (IBM Corporation, Armonk, NY, USA). Continuous variables were summarized as mean ± standard deviation (SD), and categorical variables were summarized as frequencies and percentages. Group comparisons between recovered and deceased patients were performed using the unpaired Student’s t-test for continuous variables when assumptions of normality and homogeneity of variance were reasonably met, and the Chi-square test or Fisher’s exact test for categorical variables, as appropriate. A *p*-value < 0.05 was considered statistically significant. Treatment-related information is presented descriptively only; no comparative effectiveness analysis or severity-adjusted treatment comparison was performed.

## 3. Results

### 3.1. Incidence Rate and Time Trend

**Figure 1:**
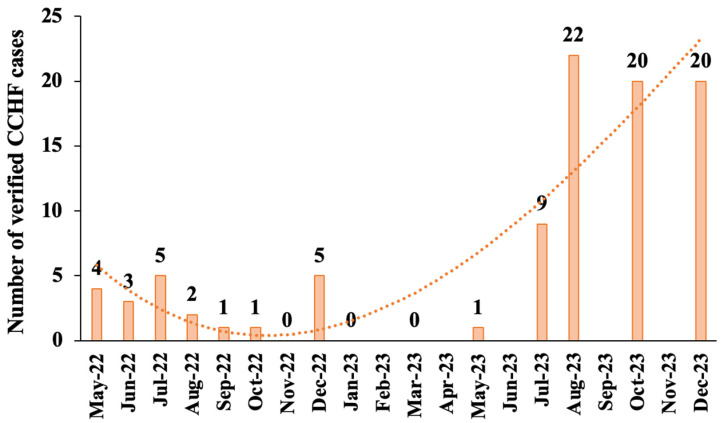
Temporal dynamics of PCR-confirmed CCHF cases in 2022–2023. The y-axis represents the number of verified CCHF cases. The x-axis represents the chronological fit for the indicated number of cases per month. The orange dotted polynomial line is the trend line with order of 3 using Microsoft Excel version 16.104 (25121423) and is shown for descriptive purposes only.

In 2022, the CCHF incidence rate in Iraq was 0.7 per 100,000 individuals. However, in 2023, the incidence rate rose notably to 2.4 per 100,000 individuals. The temporal distribution of reported CCHF cases in 2022 indicated that the first case in Basrah occurred in May, with the highest monthly case counts observed from May to July, followed by a secondary increase in December. Notably, the incidence recorded in May was comparable to that in July and December 2022 (Figure 1). In 2023, the noticeable rise in CCHF cases showed a temporal progression that spanned from May to December (Figure 1). The data indicates a noticeable rise in cases beginning in May 2023, with a peak of 22 reported in August, followed by a slight decline to a rate of 20 cases per month in October (Figure 1).

### 3.2. Sociodemographic and Epidemiological Characteristics

The average age of the patients was 35.08 ± 14.59 years, with a higher percentage of males (55.7%). Most participants (88.5%) were from the Basrah governorate, and 80.3% originated from rural regions. Self-employed represented the highest percentage 49.2% followed by housewives, 42.6% (Table 1).

A significant proportion of the participants (72.1%) were documented to have prior animal interactions, with around 59% reporting regular or frequent animal contact, particularly through slaughtering activities. Interestingly, 4.9% of participants reported a previous encounter with rodents, and 9.8% disclosed either an index case within their families or a prior experience of direct contact with CCHF patients, and 3.3% of the patients reported traveling outside Iraq (Table 2).

### 3.3. Clinical Characteristics of CCHF Disease

**Figure 2:**
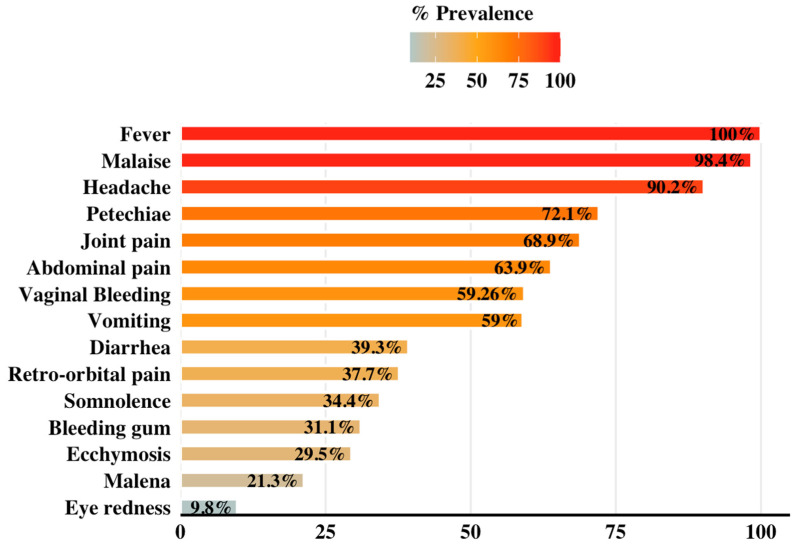
Prevalence of clinical symptoms in patients with Crimean–Congo hemorrhagic fever (CCHF). The horizontal bar plot shows the percentage prevalence (%) of individual symptoms, ranked from highest to lowest prevalence. The bars are colored on a diverging scale (skyblue to red) with a midpoint at 50%, reflecting symptom frequency distribution across cases. The data labels (% values) are positioned within bar ends for clarity. The plot was generated using RStudio software version 2025.09.2 Build 418 (Posit, Boston, MA, USA).

All the patients in this study presented with fever, while 98% reported generalized weakness and malaise. Neurological problems were common, with 90.2% of the individuals reporting headaches and 35.4% reporting somnolence (Figure 2). A high proportion of the patients also exhibited gastrointestinal problems, with abdominal discomfort (63.9%), vomiting (59%), and/or diarrhea (39.3%). Bleeding signs and symptoms were variable, including petechiae (72.1%) and ecchymosis (29.5%), as well as gingival bleeding (31.1%), melena (21.3%), and vaginal bleeding in 59.2% of the women affected (Figure 2, also Table S1).

### 3.4. Clinical Laboratory Characteristics of CCHF Disease

A comparative analysis of laboratory parameters was conducted between the initial presentation and the time of discharge. The hemoglobin levels revealed a significant decrease upon discharge (*p* < 0.01); on the other hand, the other parameters of the complete blood count showed considerable improvement. The platelet count at admission was 29.45 ± 17.79, increasing to 90.98 ± 46.48 on discharge (*p* < 0.01). Bleeding profiles, including PT, aPTT, and INR measures, improved upon discharge; neither PT nor INR showed a significant change. However, the most notable improvement was observed in aPTT, which decreased from around 43.86 ± 39.75 to approximately 29.26 ± 7.95 (*p* < 0.05) (Table 3).

### 3.5. Biochemical Profile of CCHF Disease upon Admission and Discharge

A significant decrease in C-reactive protein (CRP) levels was reported upon discharge (*p* < 0.01), although no substantial drop was observed in erythrocyte sedimentation rate (ESR). Assessment of renal functions showed that serum creatinine levels, not blood urea (B. urea), decreased considerably upon discharge compared to the increased level at admission (1.4-fold, *p* < 0.05). Assessment of liver functions showed that both ALT and AST levels were around ten-fold higher than the normal range (10–40 U/L and 10–30 U/L respectively). However, both levels were substantially reduced at discharge compared to the initial admission values (*p* < 0.01 for AST and *p* < 0.05 for ALT). Conversely, no significant alterations were observed in total bilirubin or alkaline phosphatase levels (Table 4).

### 3.6. Treatment and Therapies

Depending on the severity of symptoms, the patients received various medical interventions throughout hospitalization, including supportive therapy, antibiotics, parenteral steroids, and ribavirin as antiviral therapy. Approximately 71% of the individuals had platelet transfusions, 21% received fresh frozen plasma, and 10% needed packed red blood cell transfusions (Table 5). The therapeutic approach was stratified based on severity, hence the different ratios of therapeutic modalities.

### 3.7. Hospitalization and Disease Outcome

**Figure 3:**
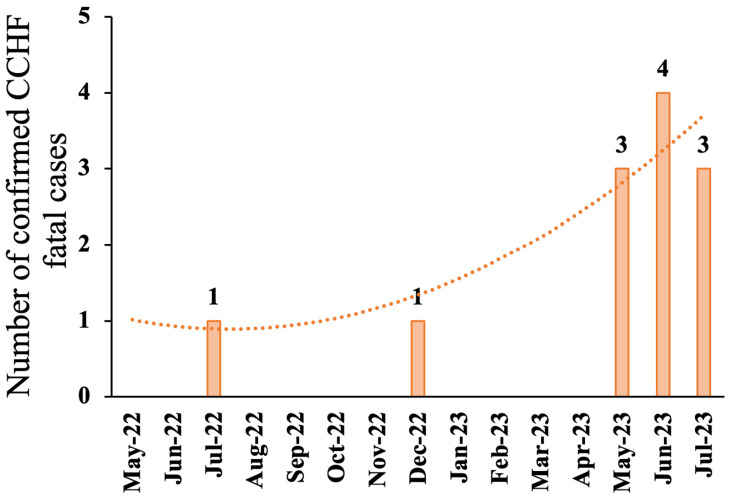
Temporal trend for mortality of PCR-confirmed CCHF cases. The y-axis represents the number of verified CCHF cases. The x-axis represents the chronological fit for the indicated number of cases per month. The orange dotted polynomial line is the trend line with order of 2 using Microsoft Excel version 16.104 (25121423) and is shown for descriptive purposes only.

In 2022, 21 patients were identified, two of whom died. The cause-specific mortality rate was calculated to be 0.07 per 100,000 person and the case fatality ratio was 9.5%. From the beginning of 2023 until 1 August 2023, the cause-specific mortality rate increased to 0.3 per 100,000 persons, and the case fatality ratio rose significantly to 13.9% over the reported period (Figure 3).

Further analysis of the research cohort showed that about 83.6% of the patients displayed better outcomes compared to the typically reported CCHF mortality, which could exceed 30% [28]. These patients were discharged in clinically stable conditions after an average hospitalization of 5.56 days. Most patients (82%) were discharged within a week after admission.

### 3.8. The Risk Factors Associated with Extended Hospitalization

We identified a significant association between a history of animal contact, lower levels of hemoglobin and platelets, higher levels of CRP, and an increased likelihood of prolonged (>7 days) hospitalization (*p* < 0.05). There was no significant association between extended hospitalization and the following factors: age, sex, history of contact with patients with CCHF, history of an index case in the family, higher WBC count, lower lymphocyte count, higher neutrophil count, and higher ESR level (*p* > 0.05) (Table 6 and Table S2).

### 3.9. Predictors of Mortality

**Figure 4:**
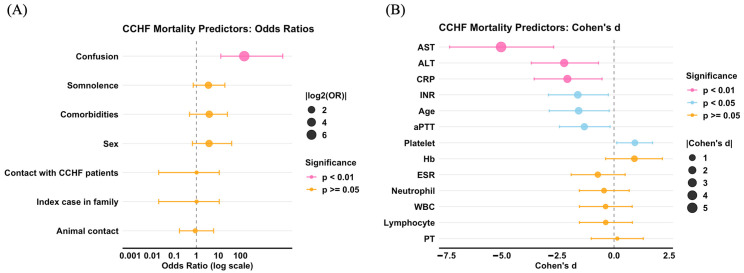
Predictors of mortality in Crimean–Congo hemorrhagic fever (CCHF) patients: (**A**) Forest plot of odds ratios for categorical predictors of mortality versus recovery in 61 patients, using summary-level 2 × 2 comparisons from 51 recovered and 10 deceased cases. Points represent odds ratios with horizontal confidence intervals, plotted on a logarithmic scale with a dashed reference line at OR = 1. Point size is proportional to effect magnitude $|{log}_{2}(OR)|$, and colors indicate statistical significance: hot pink for *p* < 0.01, sky blue for *p* < 0.05, and orange for *p* ≥ 0.05. The rat/rodent exposure row was removed from the plot because its odds ratio was not estimable on the log scale due to a zero cell count. (**B**) a forest plot of Cohen’s d for continuous clinical and laboratory variables comparing recovered and deceased patients, with horizontal confidence intervals around each estimate. Point size is proportional to ∣d∣, and the same color scheme indicates significance. Variables include age, hemoglobin, WBC, lymphocyte percentage, neutrophil percentage, platelet count, CRP, ESR, ALT, AST, PT, aPTT, and INR, with units shown in the axis labels or caption as applicable. Categorical variables include sex, comorbidities, animal contact, rat/rodent exposure, contact with CCHF patients, index case in family, somnolence, and confusion. RStudio software version 2025.09.2 Build 418 (Posit, Boston, MA, USA) using ggplot2.

There was a strong association between age (*p* < 0.05), decreased platelet counts (*p* < 0.05), elevated CRP (*p* < 0.01), aPTT (*p* < 0.05), INR (*p* < 0.05), both elevated ALT and AST (*p* < 0.01), and the presence of confusion and an increased risk of mortality (Figure 4). There was no observed association between sex or comorbidities and an elevated risk of death. Moreover, there was no apparent association between the history of animal contact, family history of the index case, history of contact, or probable way of transmission and higher risk of death. In addition, no significant correlation was observed between white blood cell count, differential erythrocyte sedimentation rate (ESR) results, and an elevated risk of death (Table 7).

## 4. Discussion

Consistent with published data [23,24], our results showed that the incidence of CCHF-reported cases is increasing in Iraq, particularly in the southern provinces [25]. During 2022 and 2023, CCHF cases peaked in July (2022) and August (2023). The reported increase in the number of CCHF cases during winter in 2022 and 2023 is not common. The increase in CCHF cases during the winter months was unusual. Although we did not measure environmental variables, prior studies suggest that climatic and ecological changes may affect tick distribution and CCHF seasonality; thus, warmer winters may be a possible contributing factor rather than a proven cause [29,30]. The data showed a CFR of 9.5%, which is slightly higher than the study by Khafaji et al. [20] but lower than the study by Al Salihi et al. [31], which reported about 13%.

In the present study, the prevalence of CCHF was higher among males, individuals with limited access to the education system, rural areas, housewives, and individuals with a history of animal contact, such as breeders or butchers. These results are consistent with previous studies [32,33]. Occupational activities are important risk factors for contracting CCHF. However, housewives constitute high percentages of CCHF-confirmed cases in several studies. This could be attributed to direct contact with viremic meat when preparing food or caring for CCHF patients at home. Our results also support that most infected people were in contact with infected animals rather than tick bites. In Iraq, sheep have the highest incidence of CCHF at 57%, followed by goats at 50% and cattle at approximately 30% [34], making livestock a likely transmission route in the country.

Interestingly, our data revealed that about 5% of CCHF confirmed cases had a history of contact with rodents, suggesting the possibility that rodents might contribute to the circulation of CCHFV in the population. Future studies examining viral sequences derived from rodents and humans in the same geographic region will help to address this issue. Both anti-CCHFV antibodies and viral RNA were detected in about 6.5% of the screened rodents in Kenya [35]. However, this finding is based on a limited number of cases and does not provide sufficient evidence to support a causal or epidemiologically significant role for rodents in CCHFV transmission, but it supports further studies aimed at assessing the role of rodents in CCHFV circulation. Our findings also support the idea that multiple contacts with animals can critically contribute to human transmission. Several cultural and occupational practices in Iraqi communities may facilitate the emergence of familial or occupational CCHF clusters. In particular, shared work environments among relatives—such as collective involvement in butchering or animal breeding—can increase the likelihood that an index case arises within a family unit and subsequently exposes other members. In addition, infection prevention and control measures in Iraq are affected by cultural opposition to isolation of infected family members, which facilitates exposure to contaminated food and body fluids [36,37].

Dominant symptoms were those previously described [38], including fever, malaise, headache, and abdominal pain. Clinical presentation and outcome of CCHFV infection result from a combination of viral and host determinants. CCHFV Asia 1 strain is dominant in Iran, Pakistan, and Iraq [39,40], and is associated with clinical features similar to those in EMR. Vaginal bleeding is one of the uncommon gynecological symptoms associated with this infection, and with other constitutional signs and symptoms, could be a suggestive sign of CCHF [16]. Hemorrhage, hematemesis, ecchymosis, gingival bleeding, melena, and somnolence are significantly more common in cases that lead to fatality.

Joint pain is a common consequence of viral infection [41]. CCHFV activates CD4+ T-helper cells, releasing cytokines that cause macrophage activation, thus promoting joint inflammation. However, joint pain is not a feature of the clinical picture of CCHF, but inflammatory changes associated with CCHFV infection can cause blood vessels damage and vasodilation, leading to coagulation, hemorrhage, and organ failure [42,43]. Retro-orbital pain and eye redness were reported in 37.7% and 9.8% of the patients, consistent with a Turkish study, where 73% suffered from ocular symptoms accompanied by long prothrombin time [44].

Survived patients showed significant improvements in platelet count, aPTT, and INR at the time of discharge. These parameters are critical prognostic factors that discriminate between fatal and non-fatal cases of CCHF. Although statistically no significant (*p* value = 0.937), differences in PT value were noticed between the fatal and non-fatal cases, in which both showed significantly elevated PT values (~17 s) compared to discharged values (Table 4) and normal values (9–13 s). Future research and investigation are needed to address and stratify elevated PT parameters between fatal and non-fatal groups to understand the root cause of such observations.

Hence, an extreme decrease in platelet counts and an increase in aPTT can determine the disease outcome [45]. We observed elevated AST and ALT in agreement with studies by Rathore et al. and Amin et al. [46,47]. Our findings suggest that liver insult, reflected by elevated AST and ALT, is a predictor of a fatal outcome of CCHF. This observation aligns with global meta-analyses of CCHF clinical parameters by Khafaji et al., which delineated four domains that significantly differentiate between survival and non-survival groups: platelet disorders, coagulopathies, fibrin disorders, and liver injury [48]. In addition, we found that confusion and elevated CRP were also predictors for mortality, which is consistent with published studies [49,50,51,52]. However, Oygar et al., in 2023, found that low CRP values raise suspicion of CCHF in endemic regions [53].

We found decreased serum creatinine levels upon discharge, and its elevation above 1.4 mg/dL has been shown to reduce the probability of surviving CCHF [54], suggesting that the magnitude of kidney dysfunction is a predictor of CCHF outcome. However, a study showed that age was one of the clinical variables with no significance difference between fatal and non-fatal cases [49].

Discharge, upon recovering normal clinical parameters, of more than 82% of the admitted CCHF cases reflects improved management of CCHF cases in Basrah compared to the reported 30% CFR in different geographical areas [28]. All the patients received treatment for associated symptoms, including acetaminophen as an antipyretic and omeprazole as a proton pump inhibitor to prevent gastrointestinal bleeding [43,55]. Broad-spectrum antibiotics were used in about 35% of the cases, a common practice to prevent secondary or concomitant infections including herpes simplex, urinary tract infection, rheumatoid fever and Streptococcus pyogenes [56,57]. In this study, management records indicate that severe cases requiring tracheal intubation or urinary catheterization were also given antibiotics as prophylactics to avoid bacterial secondary infection, or cases that developed high-grade fever and leukocytosis with positive culture for certain Gram-negative bacteria. To improve hematological recovery and to reduce the need for platelet transfusion, corticosteroids were used in more than 70% of the diagnosed patients as a first line of treating low platelet count [58,59]. The antiviral ribavirin was used to treat patients with confirmed CCHFV infection according to WHO recommendations, and studies showed a decreased mortality rate associated with its use. However, ribavirin efficacy is still debatable and needs more clinical trials [60,61]. The bleeding tendency was one of the factors that prolonged the hospitalization time; therefore, the patients were asked to stop anti-platelet factors such as Aspirin before the blood test, as it interferes with the coagulation mechanism. Most patients were committed to the treatment protocol and hospitalization time, and roughly <1% were left on their own responsibility, but records indicate that a higher percentage were left when asked to do RT-PCR, which might increase CCHF prevalence if included. Such a decision by patients could be categorized as a refusal bias due to being intimidated as people infected with deadly diseases, which could impact their psychological and mental status, or being exposed to infected others [62].

The study has several limitations. The data came only from the Basrah Governorate, which does not necessarily represent the clinical management in other parts of Iraq, particularly the mid and northern governorates. Several clinically important predictors, including viral load, time from symptom onset to hospitalization, and timing of ribavirin initiation, were not consistently available in the retrospective records and were therefore not included in the analysis. Their absence may have limited the completeness of risk-factor assessment and may have influenced the estimation of mortality predictors. Selection bias should be considered when interpreting these findings. Because this study was conducted in a single hospital-based infectious diseases unit, the included patients may not fully represent all CCHF cases in the region. Referral patterns and illness severity may have influenced which patients were admitted and recorded in the dataset, introducing referral bias. In addition, confounding by indication may have affected treatment-related observations, as therapeutic decisions were likely influenced by disease severity and clinical condition. Therefore, the associations reported in this study should be interpreted with caution as observational findings rather than evidence of causality. Future multi-center prospective studies with standardized severity assessment are needed to better address these sources of bias.

## 5. Conclusions

There is an increase in CCHF-confirmed cases in southern Iraq, with an increased CFR in 2023. We used a battery of clinical features and laboratory tests to evaluate suspected and confirmed cases of CCHF, and identified several predictors of mortality, including increasing age, decreased platelet counts, elevated CRP, aPTT, INR, ALT, and AST (admission vs. discharge times) and confusion. Proper and timely intervention aimed at improving the mentioned parameters can help to manage CCHF cases, especially under the current absence of specific treatment and vaccines. Better and broader awareness should be initiated with a focus on applying IPC measures such as wearing gloves, avoiding contact with the blood of infected people and livestock, and avoiding nosocomial infections. Identification of subclinical cases is critically important as they can contribute to viral spread and can develop clinical symptoms at any time. This underscores the need for effective surveillance protocols in highly endemic areas. This study highlights the need for policymakers to map procedures to control the spread of CCHF. Developing therapeutics and vaccines, as well as antibody-based therapies, is of critical importance. The study also necessitates the need to apply a one-health approach to CCHF surveillance and cross-sectoral collaboration (physicians, veterinarians, entomologists and environmental scientists) to improve prevention strategies in risk areas.

## Figures and Tables

**Table 1:** Sociodemographic characteristics of the patients (*N* = 61).

Sociodemographic Characteristics		Frequency (%) (*N* = 61)
**Age/Years**	Mean ± SD	35.08 ± 14.59
	Range	12–75
**Gender**	Male	34 (55.7%)
	Female	27 (44.3%)
**Governorate**	Basrah	54 (88.5%)
	Missan	6 (9.8%)
	Thi-Qar	1 (1.7%)
**Residency**	Rural	49 (80.3%)
	Urban	12 (19.7%)
**Educational level**	Read and write	17 (27.8%)
	Primary school	30 (49.2%)
	Secondary school	10 (16.4%)
	College	4 (6.6%)
**Employment status**	Housewives	26 (42.6%)
	Employed (Officer/Teacher)	3 (4.9%)
	Self-employed (Butchers/Farmers)	30 (49.2%)
	Other (Retired teacher/Students)	2 (3.3%)
**Smoking status**	Non-smoker	40 (65.6%)
	Smoker	21 (34.4%)
**Comorbidities**	Negative	53 (86.9%)
	Positive	8 (13.1%)

Background highlight and bold text were retained for readability.

**Table 2:** Epidemiological characteristics of the patients (*N* = 61).

Epidemiological Characteristics (Past History)		Frequency (%) (*N* = 61)
**Animal contact**		44 (72.1%)
**Multiple exposure to animals**		26 of 44 (59.09%)
**Exposure to rats/rodents**		3 (4.9%)
**Contact with CCHF patients**		6 (9.8%)
**Index case in family**		6 (9.8%)
**Travel**		2 (3.3%)
**Mode of transmission**	**Animal contact**	38 (62.3%)
	**Tick-bite**	14 (23.0%)
	**Unknown**	9 (14.7%)

Multiple exposure: The same person was exposed to different animals. Background highlight and bold text were retained for readability.

**Table 3:** The complete blood count (CBC) on admission and discharge of the patients (*N* = 61).

Variables		On Admission	On Discharge	*p*-Value *
**CBC**	**Hb (g/dL)**	11.86 ± 2.47	11.12 ± 2.17	0.001
	**WBC (×10^9^/L)**	4.49 ± 3.11	5.86 ± 2.19	0.001
	**Lymphocyte (%)**	30.43 ± 10.84	35.79 ± 12.06	0.002
	**Neutrophil (%)**	57.66 ± 15.03	51.56 ± 13.03	0.009
	**Platelet (×10^9^/L)**	29.39 ± 23.61	90.98 ± 46.48	0.001
**Bleeding profile**	**PT (seconds)**	29.45 ± 17.79	12.46 ± 4.18	0.233
	**PTT (seconds)**	43.86 ± 39.75	29.26 ± 7.95	0.019
	**INR**	1.22 ± 0.35	1.09 ± 0.28	0.053

* *p*-value for paired sample student *t*-test. Background highlight and bold text were retained for readability.

**Table 4:** Biochemical profile on admission and discharge of the patients (*N* = 61).

Variables		On Admission	On Discharge	*p*-Value *
**Inflammatory**	**CRP (mg/L)**	13.06 ± 12.41	5.62 ± 5.11	0.001
**Markers**	**ESR (mm/hr)**	35.21 ± 23.02	33.04 ± 21.98	0.589
**Renal function test**	**B. urea (mg/dL)**	32.63 ± 12.77	32.71 ± 9.35	0.951
	**S. Creatinine (mg/dL)**	0.82 ± 0.45	0.68 ± 0.25	0.038
**Liver function test**	**Total S. Bilirubin (mg/dL)**	1.06 ± 0.49	1.17 ± 0.64	0.284
	**ALT (U/L)**	445.74 ± 358.07	233.15 ± 198.99	0.036
	**AST (U/L)**	477.07 ± 465.72	184.79 ± 161.52	0.001
	**ALP (IU/L)**	138.57 ± 82.04	115.47 ± 84.49	0.144

* *p*-value for paired sample student *t*-test. Background highlight and bold text were retained for readability.

**Table 5:** Treatment received by the patients during the hospitalization.

Treatment	Frequency/Percentages
Supportive treatment (IV Fluid, paracetamol, omeprazole)	61 (100%)
Broad spectrum antibiotics	21 (34.42%)
Steroids (Dexamethasone)	45 (73.77%)
Ribavirin	61 (100%)
Platelets transfusion	20 (71.4%)
Fresh frozen plasma	6 (21.4%)
Blood transfusion	3 (10.7%)

Background highlight were retained for readability.

**Table 6:** Risk factors and their statistical association with prolonged hospitalization.

Variables	≤7 Days	>7 Days	*p*-Value
**Age (years)**	30.27 ± 16.38	36.14 ± 14.12	0.229 *
**Sex**			0.450 $
Male	29 (85.3%)	5 (14.7%)	
Female	21 (77.8%)	6 (22.2%)	
**Comorbidities**			0.627 ^
Positive	6 (75.0%)	2 (25.0%)	
**History of animal contact**	11 (25.0%)	33 (63.6%)	0.025 $
**History of exposure to rats/rodents**	0 (0.0%)	3 (100.0%)	0.626 ^
**History of contact with patients of CCHF**	0 (0.0%)	6 (100.0%)	0.580 ^
**History of index case in family**	0 (0.0%)	6 (100.0%)	0.112 ^
**Mode of transmission**			0.244 ^
Animal contact	29 (76.3%)	9 (23.7%)	
Tick-bite	12 (85.7%)	2 (14.3%)	
Unknown	9 (100.0%)	0 (0.0%)	
**Severity score index**	5.63 ± 2.72	6.01 ± 4.00	0.888 *
**Complete blood count**			
Hb (g/dL)	12.50 ± 2.24	10.51 ± 2.72	0.041 *
WBC (×10^9^/L)	4.18 ± 2.96	6.15 ± 4.09	0.156 *
Lymphocyte (%)	32.10 ± 11.79	25.18 ± 9.96	0.060 *
Neutrophil (%)	56.95 ± 15.49	57.27 ± 15.79	0.938 *
Platelet (×10^9^/L)	30.68 ± 21.08	19.23 ± 9.52	0.014 *
**Inflammatory markers**			
CRP (mg/L)	8.14 ± 4.82	26.68 ± 20.87	0.001 *
ESR (mm/hr)	27.48 ± 20.02	38.77 ± 24.42	0.221 *

* Unpaired sample student *t* test, ^ Fisher exact test, $ Chi-square test. Background highlight and bold text were retained for readability.

**Table 7:** Predictors of mortality from CCHF.

Variables	Recovered (n = 51)	Dead (n = 10)	*p*-Value for Group Comparison
**Age (years)**	32.57 ± 12.71	47.90 ± 17.39	0.023 *
**Sex**			0.162 ^
Male	26 (50.90%)	8 (80.0%)	
Female	25 (49.10%)	2 (20.0%)	
**Comorbidities**			0.115 $
Positive	5 (9.8%)	3 (30.0%)	
**History of animal contact**	37 (72.5%)	7 (70.0%)	1.000 $
**History of exposure to rats/rodents**	3 (5.8%)	0 (0.0%)	0.646 ^
**History of contact with patients with CCHF**	5 (9.8%)	1 (10.0%)	1.000 ^
**History of index case in family**	5 (9.8%)	1 (10.0%)	1.000 ^
**Mode of transmission**			0.422 $
Animal contact	33 (64.7%)	5 (50.0%)	
Tick-bite	10 (19.6%)	4 (40.0%)	
Unknown	8 (15.7%)	1 (10.0%)	
**Complete blood count**			
Hb (g/dL)	13.17 ± 1.51	11.94 ± 2.54	0.052 *
WBC (×10^9^/L)	4.39 ± 3.06	5.27 ± 4.20	0.544 *
Lymphocyte (%)	30.33 ± 10.96	33.50 ± 15.41	0.548 *
Neutrophil (%)	56.29 ± 15.31	60.70 ± 16.22	0.442 *
Platelet (×10^9^/L)	30.58 ± 23.69	18.60 ± 11.84	0.024 *
**Inflammatory markers**			
CRP (mg/L)	13.63 ± 15.22	44.51 ± 28.40	0.007 *
ESR (mm/hr)	22.00 ± 15.80	30.76 ± 21.70	0.176 *
**Liver function test**			
ALT (U/L)	372.22 ± 154.07	644.80 ± 222.75	0.001 *
AST (U/L)	475.92 ± 143.01	978.80 ± 171.35	0.001 *
**Bleeding profile**			
PT (seconds)	17.35 ± 3.02	17.02 ± 4.00	0.937 *
aPTT (seconds)	36.16 ± 17.79	49.90 ± 15.15	0.026 *
INR	1.20 ± 0.34	1.61 ± 0.45	0.027 *
**Neurological complications**			
Somnolence	15 (29.4%)	6 (60.0%)	0.062 $
Confusion	1 (1.9%)	8 (80.0%)	0.001 ^

* Unpaired sample student *t* test. Background highlight and bold text were retained for readability. ^ Fisher exact test. $ Chi-square test.

## Data Availability

The original contributions presented in this study are included in the article.

## Supplementary Materials

The following supporting information can be downloaded at https://www.mdpi.com/article/10.3390/idr18030049/s1. Table S1: Clinical presentations of the patients (*N* = 61). Table S2. Duration and outcome of hospitalization.

## Abbreviations

The following abbreviations are used in this manuscript:

CCHF	Crimean–Congo hemorrhagic fever
CCHFV	CCHF virus
PCR	polymerase chain reaction
EMR	Eastern Mediterranean Region
PT	prothrombin time
PTT	partial thromboplastin time
aPTT	activated partial thromboplastin time
ALT	alanine aminotransferase
AST	aspartate aminotransferase
SSI	severity score index
INR	international normalized ratio
CRP	C-reactive protein
ESR	erythrocyte sedimentation rate
