# Supplementary material for: Clinical Characteristics, Risk Factors, and Predictors of Fatal Outcomes and Prolonged Hospitalization of Crimean–Congo Hemorrhagic Fever Cases in Basrah, Iraq
Source: Infect Dis Rep. 2026 May 19;18(3):49. doi: 10.3390/idr18030049 (PMC13214867; doi:10.3390/idr18030049)
Supplement: Supplementary file 1 [file idr-18-00049-s001.zip › idr-4214888-supplementary.pdf]

Table S1. Clinical presentations of the patients (*N*=61).

The clinical presentation	Frequency (%) (N = 61)
Fever	61 (100.0)
Headache	55 (90.2)
Malaise	60 (98.4)
Somnolence	21 (34.4)
Joint pain	42 (68.9)
Retro-orbital pain	23 (37.7)
Eye redness	6 (9.8)
Abdominal pain	39 (63.9)
Vomiting	36 (59.0)
Diarrhea	24 (39.3)
Bleeding gum	19 (31.1)
Petechiae	44 (72.1)
Ecchymosis	18 (29.5)
Malena	13 (21.3)
Vaginal Bleeding	16 of 27 (59.26%)

Table S2. Duration and outcome of hospitalization.

<table>
<tr><th colspan="2">Outcome and Hospitalisation</th><th>Frequency (%) (N = 61)</th></tr>
<tr><td rowspan="2">Outcome at discharge</td><td>Alive</td><td>51 (83.6%)</td></tr>
<tr><td>Died</td><td>10 (16.4%)</td></tr>
<tr><td rowspan="3">Duration of hospitalisation</td><td>Mean ± SD / Range</td><td>5.56 ± 2.20 / (1-11 days)</td></tr>
<tr><td>≤ 7 days</td><td>50 (82.0%)</td></tr>
<tr><td>> 7 days</td><td>11 (18.0%)</td></tr>
</table>
